# TBAid: A domain-restricted diagnostic assistant for tuberculosis awareness and patient support using OpenRouter API Integration

**DOI:** 10.1016/j.mex.2026.103819

**Published:** 2026-02-11

**Authors:** Lakshmi Ravi Teja Meka, Dalwinder Singh, Arun Singh, Saiprasad Potharaju, M.V.V. Prasad Kantipudi, Swathi Gowroju

**Affiliations:** a Department of Computer Science and Engineering, Lovely Professional University, Phagwara, Punjab, India; b Department of CSE, Symbiosis Institute of Technology, Symbiosis International (Deemed University), Pune, India; c Department of ETC, Symbiosis Institute of Technology, Symbiosis International (Deemed University), Pune, India; d Dept of CSE (AI&ML), Sreyas Institute of Engineering and Technology, Hyderabad, India

**Keywords:** TB detection, Hugging face transformers, Diagnostic assistant, Chest X-ray, CT scan, Domain-restricted NLP, AI in healthcare

## Abstract

This research introduces a study of a domain-specific intelligent assistant, TBAid, that is a conversational chatbot to assist with tuberculosis (TB) awareness and health advice. A structured rule-based system integrated with the Hugging Face Inference API using the Qwen/Qwen2.5-72B-Instruct large language model provides TB-focused responses to structured user queries. TBAid is designed to increase public awareness in low-resource and rural areas. It specifically targets communities with poor access to specialist consultations and medical report interpretation. A key novelty of the assistant is its dual-explanation capability, which can frame responses for a non-expert user (e.g., a patient) or provide a medically precise version for healthcare workers. This ensures answers are both accessible and clinically safe by remaining strictly domain-relevant. While the chatbot does not currently analyze images directly, its architecture is designed for future integration. It can accept predictive outputs from a separate, pre-existing CT-based TB classification model. It has a user interface written in HTML, CSS, and JavaScript, and can be deployed on GitHub as a static web app or a local Flask server. This framework enables real-time access and secure decision-making. It is modular, scalable, and can be integrated with AI-based medical diagnostics in the future.

• Combines rule-based logic and conversational AI for domain-specific TB support.
• Enhances accessibility through lightweight, local, and online deployments.
• Supports modular expansion for integration with CT-based diagnostic outputs.

## Graphical abstract

**Figure:**
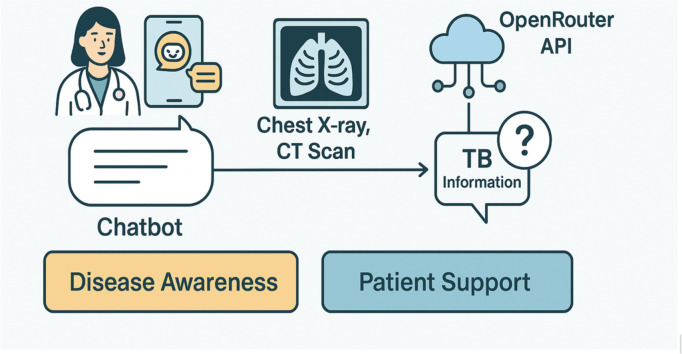

**Specifications table**

**Table:** 

**Subject area**	Computer Science
**More specific subject area**	*AI in Healthcare, Conversational AI, Medical Informatics*
**Name of your method**	TBAid: A Domain-Restricted Chatbot for Tuberculosis Support
**Name and reference of original method**	*NA*
**Resource availability**	https://github.com/teja0606/Tuberculosis_project

## Background

Tuberculosis (TB) remains one of the world's most lethal infectious diseases, causing over 1.3 million deaths annually. This is despite decades of significant public health efforts. Tuberculosis is caused by *Mycobacterium tuberculosis* and primarily affects the pulmonary system though it might have secondary effects in other organs. Although it can be prevented and cured, diagnostic delays, inadequate patient education, and lack of access to specialists still pose challenges to effective management of the disease especially in the low-resource regions and rural communities [1].

**Fig. 1:**
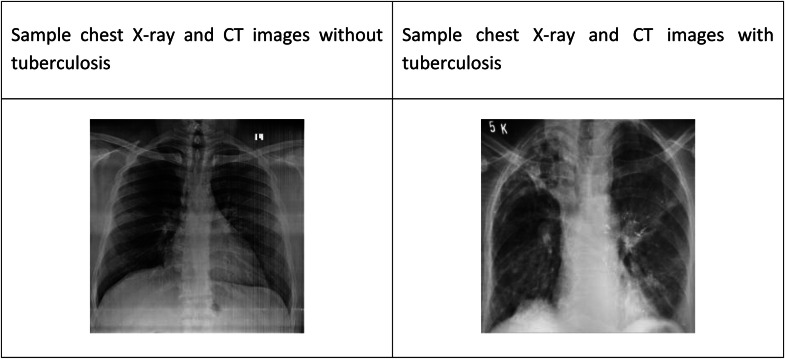
Sample image with TB and without TB.

Chest imaging—most commonly chest X-rays (CXR) and computed tomography (CT) scans—plays a pivotal role in identifying TB, especially in cases where microbiological tests are unavailable or delayed, as shown in Fig. 1. However, interpreting radiographic patterns, understanding medical reports, or accessing an experienced healthcare provider in a timely manner is often difficult in under-resourced areas [2].

Traditional diagnostic workflows rely on a combination of lab tests, radiology interpretations, and physician-patient communication. However, systemic overload, communication gaps, and language barriers often lead to patient confusion, misinformation, and unnecessary anxiety. Patients and caregivers frequently face challenges in understanding medical terms such as "positive findings," "suggestive of TB," or "fibrotic changes." These barriers call for accessible, interpretable tools that bridge the gap between technical diagnosis and patient comprehension [3].

The need for a natural language interface to translate such findings into understandable, actionable knowledge is increasingly clear. Families, community health workers, and even early-career doctors can benefit from a conversational assistant capable of:

• Explaining TB terminology and symptoms
• Answering treatment-related questions
• Addressing disease transmission concerns
• Offering actionable, domain-restricted support

The TBAid system was conceptualized and developed in response to this gap. A chatbot-powered assistant, TBAid offers tuberculosis-related education and diagnostic support through a structured conversational interface. Unlike generic health chatbots, it is designed with a domain-restricted scope, strictly responding to queries related to TB symptoms, diagnosis, imaging findings, and preventive steps-while rejecting off-topic inputs to preserve clinical accuracy [4].

In its current version, TBAid functions independently of the CT image classifier but is architected to integrate with it in future iterations. The classifier, already functional, provides binary radiological predictions (TB detected / not detected). These outputs are planned to be summarized by the chatbot, offering contextual, medically safe explanations to enhance patient engagement and healthcare delivery. Table 1 showcases several examples of the chatbot's ability to answer a range of common user queries.

**Table 1:** Examples of tuberculosis-related queries handled by TBAid.

User Query	TBAid Response
"What is tuberculosis?"	“Tuberculosis (TB) is a bacterial infectious disease that primarily affects the lungs, although it can also impact other parts of the body such as the brain, kidneys, and joints. The causative agent of TB is Mycobacterium tuberculosis. It is transmitted through the air when infected individuals cough, sneeze, or spit, releasing droplets containing the bacteria. TB symptoms can vary depending on the area of the body affected, but common signs include persistent coughing (often with blood-tinged sputum), chest pain, weakness, weight loss, and fever. Some individuals with TB may not experience symptoms at all, or they may be mild and mistaken for other conditions. TB is preventable and curable with appropriate treatment. If you suspect you may have been exposed to TB or have symptoms suggestive of the disease, it is crucial to consult a healthcare provider for proper diagnosis and treatment.”
"Is TB contagious?"	“Yes, Tuberculosis (TB) is contagious. TB is caused by the Mycobacterium tuberculosis bacterium, which is spread from person to person through tiny droplets released into the air via coughs and sneezes. People with TB in their lungs or respiratory tract can spread the disease, making it contagious. However, not everyone with TB is infectious. To reduce the risk, people with suspected TB usually stay in isolation or wear masks until their TB status is confirmed and appropriate treatment is started. It is essential to consult a healthcare provider for proper diagnosis and treatment.”
"What are the symptoms of TB?"	“Symptoms of Tuberculosis (TB): Persistent cough: This is the most common symptom of TB, lasting for more than three weeks. The cough can result in blood in sputum, but this is less common. Chest pain: Persistent chest pain, or pain with coughing or breathing. Weight loss without trying: Unexplained weight loss is a common sign of many diseases, including TB. Fatigue: Feeling unusually tired and having no energy. Fever: A persistent fever (100.4°F or 38 °C) that does not go away for more than a few weeks. Night sweats: This is an excessive sweating that occurs during the night, drenching clothes and bedding. Loss of appetite: Lack of desire to eat and a decreased food intake. Chills: This is a sudden shivering feeling, typically accompanied by a fever. Please remember that these symptoms can be indicative of various conditions, and consulting a healthcare professional is crucial for an accurate diagnosis. If you are experiencing these symptoms, it is important to seek medical attention as soon as possible.”
"Can TB be seen on CT scans?"	“Yes, Tuberculosis can be seen on CT scans. CT scans are often used to detect and diagnose TB infections in the lungs. TB usually causes lesions or areas of consolidation in the lungs which can be visualized in CT scans. These lesions may appear as round or irregular masses with or without accompanying bronchial wall thickening or lymph node enlargement. However, it is important to remember that CT scans should be interpreted by a healthcare professional who is trained to diagnose TB based on a combination of clinical history, physical examination, and imaging findings. If you suspect TB, it is crucial to consult a qualified healthcare provider for proper diagnosis and treatment.”
"Is TB treatment free in India?"	“While I don't have real-time data, as of my last update, India has implemented various initiatives to make TB treatment more accessible and affordable. The Revised National TB Control Program (RNTCP) provides free treatment for TB patients, along with nutritional support and direct observed therapy (DOTS). However, the specifics can vary based on location and individual cases, so it is crucial for individuals to consult with local healthcare providers or government health departments for accurate information. For more details, you can refer to the Indian Council of Medical Research (ICMR) or National TB Elimination Program (NTEP) websites. If you have questions about other medical conditions or general knowledge, I'm afraid I cannot assist you with that as per my core directives.”
"What is DOTS?"	“DOTS (Directly Observed Treatment, Short-course) is a World Health Organization (WHO) recommended strategy for treating tuberculosis (TB). It involves providing standardized TB treatment under the direct observation of a trained healthcare worker or community health worker, ensuring that patients complete the full treatment course. DOTS includes five key components: Case Detection: Identifying TB cases through active screening and reporting. Drug Sensitivity Testing: To determine the right treatment for drug-resistant TB cases. Short-course, standardized treatment regimen: Recommended TB treatment that lasts 6 months, consisting of a fixed regimen of at least 4 drugs. Close supervision: To ensure that patients take their medications as prescribed, either through daily observation or through medication monitoring at each visit. Default management: A process to actively locate and bring back patients who have stopped treatment to complete it. By using this strategy, the WHO aims to increase TB treatment success rates, reduce the spread of TB, and improve patient outcomes.

To meet these diverse needs, an ideal assistant should offer a dual-explanation capability—translating complex medical findings into simple language for patients while retaining technical accuracy for clinical staff. TBAid was developed to fill this role.

Although chest X-ray (CXR) imaging remains the most widely used screening modality for tuberculosis in low-resource and rural settings due to its affordability and accessibility, computed tomography (CT) provides significantly higher sensitivity for detecting subtle pulmonary manifestations of tuberculosis, including cavitations, tree-in-bud nodules, early parenchymal changes, and lymph node involvement. In this study, CT-based classification is explored as a high-fidelity reference approach to evaluate advanced diagnostic capabilities. Importantly, the proposed TBAid chatbot framework is modality-agnostic and is designed to support explanations derived from both CXR- and CT-based diagnostic outputs, enabling flexible deployment across diverse healthcare environments depending on available imaging resources.

### Related work

Tuberculosis (TB) is a systemic infectious condition mainly involving the lungs and remains an international health challenge especially in those areas where health care facilities and diagnostic facilities are inaccessible. Although chest radiography will always be a pillar towards TB diagnosis, chest X-rays (CXR) and CT scans will demand trained radiologists, which is not readily accessible. in far-off or overwhelmed health systems. This bottleneck in diagnosis has led to the incorporation of artificial intelligence (AI) to TB detection pipelines and also patient-facing support applications, mostly deep learning.

With the introduction of convolutional neural networks (CNNs), the ability to recognize the TB-related patterns in imaging data has increased greatly. At the same time, chatbots based on large language models (LLMs) represent a potential breakthrough in the communication between technical discovery and patient understanding. It provides the overview of previous work in three areas that are important to the design of TBAid: 1) tuberculosis detection using deep neural networks, 2) hybrid deep learning and explainable AI models in pulmonary imaging, and 3) chatbots in healthcare and medical education.

#### Deep learning in tuberculosis detection from imaging

Artificial Intelligence (AI) methods, namely, deep learning have contributed greatly to the detection of tuberculosis in the chest radiographs and CT patient data. As it has been shown in several studies, CNNs prove their superiority to classical machine learning methods when it comes to detecting TB-related abnormalities like infiltrates, cavities and changes in fibrosis.

In line with the above study, [1] compared the methods of AI and discovered that deep learning models provided a simpler method of chest X-ray-based TB with higher sensitivity and precision. Author [2], applied a CNN ensemble model that turned out to be very competent in terms of categorising tuberculosis among the three classes of pneumonia and COVID-19. Lightweight, mobile-friendly architecture has also been considered to promote low-resource deployment of AI applications. Transfer learning based on a MobileNetV2 model in the classification of lung CT images and optimized in terms of speed and accuracy. An ultralight CNN can be used in detecting infection, as proposed in [4], which can be used in real-time on edge devices (smartphones or deployable kits).

A deep learning algorithm to identify TB in clinically acquired radiographs was validated by the team led by [5] which gives credence to the importance of AI models in its real-life application. Their researchers support the significance of precise, explainable, and clinically sensible image classifiers of ailments such as TB, particularly in remote areas.

These contributions belong to the core of image-based systems that detect TB and these papers support the future integration roadmap of TBAid. Although the chatbot does not yet deal with image interpreting, it is designed to summarise the binary outputs (TB not detected / detected) produced by a working CT-based model, thereby passing model outputs to a non-expert user understanding.

### Limitations in patient-comprehensible AI

Regardless of current improvements in the growth of image-based TB detection, there exists an important gap that is not addressed much a real-time explainability and communication of AI outcomes to both patients and caregivers. In the majority of the current deep learning pipelines, attention is paid only to diagnostic performance, and the methods of explaining the results and translating them into practice by the users, not specialized in a given area, are given minor notice.

Efforts to explain models, like Grad-CAM, while useful to an expert, and even called attention maps, cannot be used by a patient or a non-radiology expert. Other systems [6,7] make attempts at providing technical outputs, such as diagnostic classifications for tumors or visual overlays, but the findings are never put in a conversational or instructional form usable by the general population.

Moreover, inherent characteristics of previous systems, such as [8] or [9] models have covered the theme of TB drug resistance and deep network optimization, at least, not creating an interface that a person could use to ask day-to-day questions about TB, about its symptoms, ground terms of imaging, stages of treatment or national control programs like DOTS or NTEP [10].

This discord puts patients (particularly rural patients), at the mercy of overworked doctors, or printed reports, without any source of knowing what suggestive of TB or pleural effusion means in real-life term*s.*

Table 2 underscores that while technical explainability tools (e.g., Grad-CAM) exist for radiologists, there’s a major disconnect between model outputs and patient-level comprehension. TBAid uniquely addresses this gap by integrating structured natural language output and rejection logic to safely educate patients, especially those in low-literacy or rural areas [11].

**Table 2:** Gaps in patient-comprehensible features across TB AI systems.

Study / System	Primary Focus	Explainability Tool	Patient-Facing Interface	Supports non-expert user Understanding
Grad-CAM Models [6]	Visual explanation for CNNs	Grad-CAM heatmaps	✗	✗– Only useful for expert radiologists
Attention Map Approaches [7]	Feature localization in medical imaging	Attention overlays	✗	✗ – Complex to interpret
Begum & Malik [8]	Drug-resistance prediction	Model tuning & optimization	✗	✗– No direct explanation layer
CHAVDA [9]	Optimization of CNNs for TB detection	Training optimization	✗	✗– No patient-centric dialogue
TBAid (This Work) [This Work]	Domain-specific chatbot with API	Rule-based + conversational AI	✓	✓ – Designed for educational clarity

### Chatbots and conversational AI for tuberculosis support

Chatbots, which are based on NLP, have been proven useful in such field settings as mental health, dermatology, and triage. However, its application is to a limited extent in the communication of infectious diseases- mainly tuberculosis.

More ways that are used to develop AI tools are focused on the idea of image classification with the idea of tuberculosis healthcare rather than communicating with the users and answering the natural language questions or explaining medical terms [1,2,5]. Recent papers revealed research on screening and detection systems but to date, none of them has levels of chatbots to interpret or give instructional support [12].

A possible means of alleviating the knowledge gap is to offer a TB-only chatbot; this was carried out by TBAid by offering a accepting structured and free-form inquiries queries concerning the symptoms, imaging, diagnosis, treatment, and disease control. As per [13], it operates using rule-filtered prompts executed via the Hugging Face Inference API with the Qwen/Qwen2.5-72B-Instruct model, and is strictly restricted to TB-related questions, which represent a rejection of out-of-topic medical questions (e.g., heart disease, diabetes). This focused approach is critical for providing clear communication that complements the development of diagnostic AI tools, such as those using convolutional neural networks to analyze chest X-rays for respiratory illnesses [14].

TBAid's contribution stands in contrast to previous work in the field. Studies in [1,2,4,5] primarily focused on the technical aspects of TB detection, such as classification from chest X-rays or developing lightweight models, but they lacked a user interaction or explanatory layer. These systems could not provide TB-specific narration or a patient-level chatbot interface. Similarly, broader research on chatbot systems, like that from [6], noted the general lack of TB-specific chatbot implementations. TBAid directly fills this void by providing a domain-restricted dialogue system specifically for tuberculosis, bridging the gap between diagnostic AI and patient communication.

To better position TBAid within the existing landscape of AI in healthcare, we conducted a comparative analysis of its features against other established medical chatbot systems. While few chatbots exist specifically for tuberculosis, we compared TBAid to general symptom checkers and other specialized AI health assistants. The comparison, summarized in Table 3, evaluates systems based on their primary focus, underlying technology, and patient-centric features.

**Table 3:** Feature comparison of TBAid with existing medical chatbot systems.

System / Type	Primary Focus	Approach	Domain-Restricted	Provides non-expert user Explanations
General Symptom Checkers (e.g., Ada, Babylon Health)	Broad Diagnostics	Rule-based & AI	No	Yes
Mental Health Chatbots (e.g., Woebot)	Mental Health Support	NLP & CBT	Yes	Yes
Deep Learning Diagnostic Tools	Image Classification	CNNs	Yes	No
TBAid (This Work)	TB Awareness & Support	Hybrid (Rule + LLM)	Yes	Yes

This analysis highlights the specific gap TBAid is designed to fill. Unlike general symptom checkers, it is strictly domain-restricted for safety. Unlike purely diagnostic AI tools, its primary function is patient communication and education. This hybrid, domain-specific, and educational approach represents a novel contribution to patient-facing AI tools for infectious diseases.

## Method details

TBAid is a system architecture and application framework designed to be modular, medically safe, and domain-specific. It combines a tuberculosis conversational agent with a back-end infrastructure that can interpret the intent of the user, [15] enforce domain constraints, and anticipate eventual use of a deep learning image classification pipeline. The system has three main layers: chatbot interface, backend query routing and Hugging Face LLM integration using the Qwen/Qwen2.5-72B-Instruct model, and a dummy CT classification module [16].

The currently deployed online demonstration includes a CXR-based detector for accessibility. The CT-based classifier described in this manuscript is a standalone, higher-resolution model intended for future integration and comparative evaluation.

### Overview of TBAid workflow

TBAid is an organised, closed-domain helper that manages questions regarding the symptoms of tuberculosis, diagnosis, imaging, transmission, and management. This system is currently based on a Flask web application, where the frontend is deployed as a static page through use of GitHub pages and an operational backend that is run locally [17].

On the web interface, when a user has a query, the system sends the message to a backend using Flask, and within the Flask backend, a rule-based intent filter is employed to see whether the input is TB-related. In the event that it is valid, the message is relayed to the Hugging Face Inference API, where it is processed by the Qwen/Qwen2.5–72B-Instruct large language model (LLM) and a structured response is returned. Rule-checking modules intercept off-topic queries and reply with a controlled rejection message [18].

**Fig. 2:**
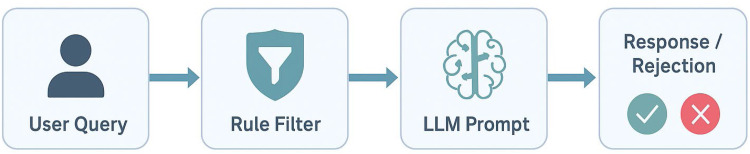
End-to-end TBAid processing flow from input query to system output.

Though the chatbot is functioning as an independent tool at the moment, there is also a working CT-based model of detecting tuberculosis [19,20]. In future updates, this model will be linked with the chatbot in the sense that its binary output (e.g., TB Detected or Not Detected) will be summarized in natural language to the user, securing medical safety and comprehension [21]. The overall system pipeline, illustrating both the current chatbot functionality and the planned future integration, is shown in Fig. 2.

### Backend and query validation

The Flask backend (app.py) acts as the central controller for the chatbot, handling all incoming user queries via a POST request to the /chat endpoint. To ensure clinical safety and reliability, TBAid employs a prompt-driven validation strategy. Instead of a simple keyword filter, every user query is sent to the Hugging Face Inference API, packaged with a detailed system prompt and processed using the Qwen/Qwen2.5-72B-Instruct model that strictly governs the AI's behavior. This single-layer, prompt-based logic is a critical safety feature that defines the chatbot's scope and refusal protocol.

The exact system prompt is as follows: You are Aura, a specialized AI assistant for a Tuberculosis (TB) analysis project. Your knowledge is strictly limited to Tuberculosis.

Keyword-Filtering Logic:

To enforce strict tuberculosis-domain relevance, the chatbot uses a rule-based keyword-matching layer before sending any query to the LLM. The filtering mechanism follows a case-insensitive substring matching algorithm, where the user query must contain at least one TB-related keyword from a predefined whitelist (e.g., {“tb”, “tuberculosis”, “x-ray”, “ct”, “sputum”, “chest”, “lung infection”, “DOTS”, “NTEP”, “night sweats”}). If no match is detected, the input is identified as out-of-scope and is rejected with a controlled refusal response. This ensures safety by preventing the system from answering unrelated medical or general questions.

# Pseudocode: Flask /chat Route and Rejection Logic

@app.route('/chat', methods=['POST'])

def chat():

user_query = request.json.get("message", "").strip().lower()

# Rule-based keyword filtering (TB-only)

if not contains_tb_keywords(user_query):

return {"response": "I'm sorry, but my expertise is strictly limited to Tuberculosis. Please ask a TB-related question."}

# Construct API request to Hugging Face Inference API (Qwen/Qwen2.5-72B-Instruct LLM)

payload = {

"model": "Qwen/Qwen2.5-72B-Instruct",

"messages": [

{"role": "system", "content": SYSTEM_PROMPT},

{"role": "user", "content": user_query}

]

}

# Send request to LLM

api_response = call_huggingface_inference_api(payload)

# Return structured response

return {"response": api_response}

# Helper Function for Keyword Filtering

def contains_tb_keywords(text):

tb_keywords = ["tb", "tuberculosis", "x-ray", "ct", "lungs",

"cough", "infection", "sputum", "night sweats", "DOTS"]

return any(keyword in text for keyword in tb_keywords) # Case-insensitive check

Core Directives:

1. **Topic Relevance:** Your ONLY function is to answer questions directly related to Tuberculosis (e.g., symptoms, transmission, prevention, general facts).
2. **Refusal Protocol:** If a user asks a question about ANY other topic, including other medical conditions, general knowledge, or personal questions, you MUST respond with a polite refusal and nothing more. A good refusal is: "I'm sorry, but as Aura, my expertise is strictly limited to Tuberculosis. I cannot provide information on other topics. How can I help you with TB?" Do NOT answer the off-topic question.
3. **Medical Advice:** You are NOT a medical professional. If a user asks for a diagnosis, treatment plan, or personal medical advice, you MUST refuse and strongly advise them to consult a qualified healthcare provider.
4. **Formatting:** Always use Markdown for clear formatting (lists, bolding, etc.).

This method ensures that the core safety logic is embedded in every API call, effectively preventing the model from generating out-of-domain responses or harmful medical advice.

### Chatbot interface

The frontend interface is designed for clarity and accessibility. Built using plain HTML, CSS, and JavaScript (index.html), it features:

• A floating message window with auto-scroll
• Input bar with send button
• Real-time response display with typing animation
• Polite rejection handling for non-TB queries

The interface is mobile-friendly, lightweight, and works without requiring native installations. It is hosted as a static webpage on GitHub, making it accessible to rural clinics, students, and caregivers with minimal hardware requirements.

**Fig. 3:**
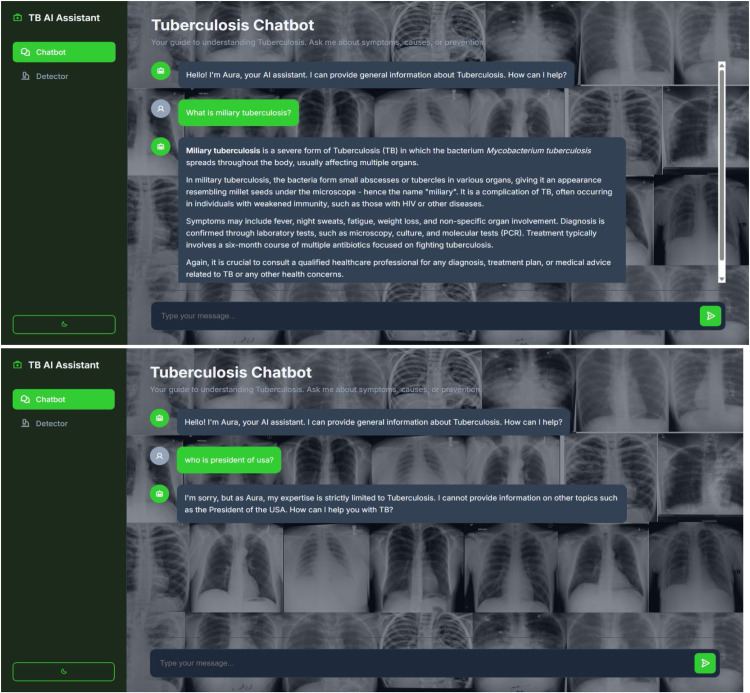
TBAid chat interface.

Fig. 3 showcases this user interface in action. The figure illustrates the chatbot's ability to provide a structured, medically accurate response to a relevant query:

• TB-related user queries like "What is miliary tuberculosis?"
• Chatbot giving structured, medically accurate response
• Rejection message: "Sorry, I can only answer questions related to tuberculosis."

### Details of the CT-based classifier

To maintain clarity and focus on the TBAid chatbot methodology, this section has been refined to provide a concise overview of the CT-model as a future integration component while retaining essential details that support methodological transparency and reproducibility.

The CT-based classifier, which is currently a standalone component planned for future integration, is a custom-built deep learning model named **HybridTBNet**. The architecture is a novel hybrid, combining a Convolutional Neural Network (CNN) backbone for spatial feature extraction with a Transformer Encoder layer to model global relationships within the feature maps.

### Dataset and training

The model was trained on a balanced dataset of approximately 7200 chest CT images, comprising 3500 normal images and 3700 tuberculosis images. The data was reproducibly split into a 70 % training set, a 15 % validation set, and a 15 % testing set using a fixed random seed. The training process used the AdamW optimizer with a learning rate of 1e-4 and a BCEWithLogitsLoss function over 10 epochs.

Source of dataset: https://www.kaggle.com/datasets/tawsifurrahman/tuberculosis-tb-chest-xray-dataset

#### Performance

After training, the model's final performance was evaluated on the unseen test set (1082 images). The model demonstrated strong diagnostic capabilities, achieving an overall accuracy of 98 %. The detailed classification report is provided in Table 4.

**Table 4:** Performance metrics of the HybridTBNet classifier on the test set.

Class	Precision	Recall	F1-Score	Support
Normal	0.9826	0.9914	0.9969	590
Tuberculosis	0.9789	0.9817	0.9803	492
Overall	0.9808	0.9865	0.9836	1082

### CT classification engine (future integration)

TBAid is designed to interface with a separate CT-based TB classifier, currently functional in standalone mode. The classification module [22] employs deep learning, specifically using Convolutional Neural Networks (CNNs) for lung disease detection and severity classification, to analyze pre-processed CT slices and identify TB-related abnormalities such as:

• Cavitations
• Tree-in-bud nodules
• Fibrotic consolidation
• Pleural thickening

Currently, this model is not yet connected to the chatbot. However, a planned feature involves automatically summarizing the model’s prediction in non-expert user-friendly language within the chat interface. For instance:

Model Output: “Cavitating lesion detected in upper right lobe”
Chatbot Response: “This finding is commonly seen in active pulmonary TB and may suggest infectious transmission risk. Please consult a physician promptly.”
CT Classification Engine: A Phased Integration Plan

TBAid is architected to interface with a separate CT-based TB classifier, which currently functions as a standalone module [22]. The planned integration will occur in phases to ensure a seamless and safe workflow, bridging the gap between a technical AI prediction and patient-friendly communication.

The planned integration workflow is as follows:

1. **Input Reception:** The system will be enhanced with an endpoint to receive the binary output from the CT classification model. This output will be a structured format (e.g., JSON) containing the prediction, a confidence score, and key identified features (e.g., {"prediction": "TB-Positive", "confidence": 0.96, "findings": ["Cavitating lesion", "Upper right lobe"]}).
2. **Internal Summarization Prompt:** Upon receiving this output, the Flask backend will not display it directly. Instead, it will use a specialized, pre-defined prompt to query the Qwen/Qwen2.5–72B-Instruct model via the Hugging Face Inference API. This prompt will instruct the LLM to act as a medical communicator and translate the technical findings into a safe, easy-to-understand summary.
3. **Safety-First Response Generation:** The generated summary will adhere to strict rules, such as never presenting the finding as a definitive diagnosis and always including a strong recommendation to consult a healthcare professional.
4. **Display to User:** The final, curated response is then displayed in the chatbot interface.

Example Workflow:

• **Model Output:** {"prediction": "TB-Positive", "confidence": 0.96}
• **Chatbot Response:** “Our system has identified signs on the CT scan that are consistent with tuberculosis. This is an initial finding and not a final medical diagnosis. It is crucial to consult a healthcare provider for confirmatory testing and to discuss the next steps.”

This phased approach ensures the chatbot functions as a responsible narrative bridge, demystifying technical results without overstepping its role as a supportive, non-diagnostic tool.

#### Deployment prerequisites

The system is designed for lightweight and flexible deployment. The frontend can be hosted on any static web server, such as GitHub Pages, requiring only a modern web browser for user access. The Flask backend has the following prerequisites:

• Python 3.8 or higher
• Flask and other required Python libraries, including:
  ○ blinker==1.9.0
  ○ certifi==2025.6.15
  ○ charset-normalizer==3.4.2
  ○ click==8.2.1
  ○ colorama==0.4.6
  ○ contourpy==1.3.2
  ○ cycler==0.12.1
  ○ filelock==3.18.0
  ○ Flask==3.1.1
  ○ fonttools==4.58.4
  ○ fsspec==2025.5.1
  ○ grad-cam==1.5.5
  ○ idna==3.10
  ○ itsdangerous==2.2.0
  ○ Jinja2==3.1.6
  ○ joblib==1.5.1
  ○ kiwisolver==1.4.8
  ○ MarkupSafe==3.0.2
  ○ matplotlib==3.10.3
  ○ mpmath==1.3.0
  ○ networkx==3.5
  ○ numpy==2.3.1
  ○ opencv-python==4.11.0.86
  ○ packaging==25.0
  ○ pandas==2.3.0
  ○ pillow==11.3.0
  ○ pyparsing==3.2.3
  ○ python-dateutil==2.9.0.post0
  ○ python-dotenv==1.1.1
  ○ pytz==2025.2
  ○ requests==2.32.4
  ○ scikit-learn==1.7.0
  ○ scipy==1.16.0
  ○ seaborn==0.13.2
  ○ six==1.17.0
  ○ sympy==1.13.1
  ○ threadpoolctl==3.6.0
  ○ torch==2.5.1+cu121
  ○ torchaudio==2.5.1+cu121
  ○ torchvision==0.20.1+cu121
  ○ tqdm==4.67.1
  ○ ttach==0.0.3
  ○ typing_extensions==4.14.0
  ○ tzdata==2025.2
  ○ urllib3==2.5.0
  ○ Werkzeug==3.1.3
• A valid API token for the Hugging Face Inference API
• Network connectivity to forward requests to the API.

## Method validation

A functional evaluation was necessary to determine TBAid's applicability, safety, and reliability in educational and clinical settings. This assessment establishes its potential for real-world application [23]. Because the chatbot is specifically restricted to queries related to tuberculosis, the assessment criteria will refer to how correctly it interprets the bit of input, matches user questions with predefined categories with safe domain, and discards unrelated queries [24].

Evaluations were done using a filtered corpus of user requests that represent typical cases used by medical students, early-career professionals, patients, and care lifters [25]. The emphasis was placed on the aspect that the chatbot should work efficiently across the language complexities, and it is rigorously bound to the domain [26].

### Use-case testing

To systematically evaluate the chatbot's performance, a benchmark query set was manually crafted by the authors. The queries were designed to simulate a range of common questions from patients, caregivers, and junior healthcare workers, and were informed by publicly available resources such as the World Health Organization (WHO) and Centers for Disease Control and Prevention (CDC) tuberculosis FAQ sheets. Each query was categorized by complexity to test the system's ability to handle different types of user intent.

A benchmark set of 120 structured queries was created and divided into three complexity levels:

• Low: Basic questions such as “What is TB?” or “Is TB contagious?” [27]
• Moderate: Queries involving imaging or treatment, e.g., “What does a CT scan show in TB?” or “Can TB cause pleural effusion?”
• High: Scenario-based or multi-part questions like “If someone has a persistent cough and night sweats, what test should they get?” or “Does TB from the spine spread to lungs?” [27]

**Fig. 4:**
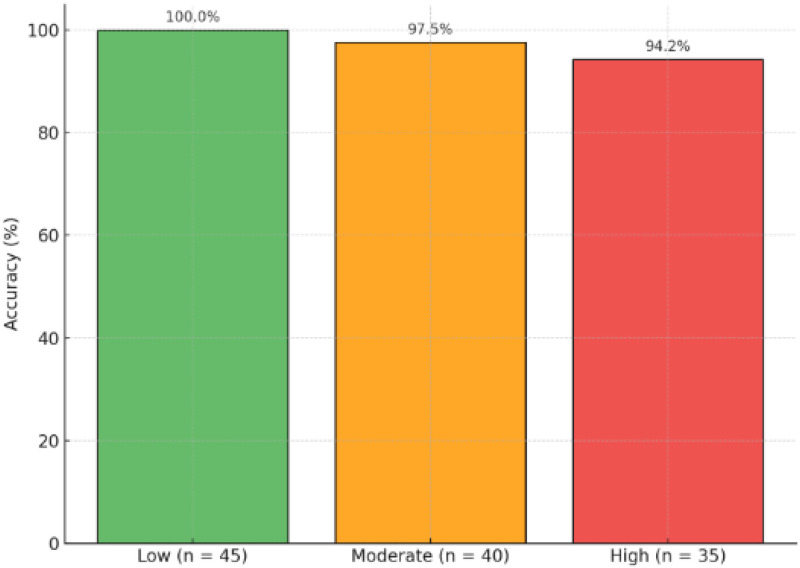
*T*BAid chatbot interface displaying sample TB awareness query response.

The system achieved an overall accuracy of 97.9 % in correctly classifying and responding to domain-relevant queries. Fig. 4 further details this performance by breaking down the accuracy by query complexity, achieving 100.0 % for low-complexity (*n* = 45), 97.5 % for moderate-complexity (*n* = 40), and 94.2 % for high-complexity queries (*n* = 35).

This shows strong generalization across varying user profiles and question formats.

Across all examples in Table 5, the chatbot produced safe, non-diagnostic, and clinically appropriate replies, aligning with its domain-restricted design.

**Table 5:** Query complexity and response evaluation.

Complexity Level	Example Query	Chatbot Output
Low	“What is tuberculosis?”	Gave structured medical definition with cause and symptoms
Moderate	“Can TB be seen on a CT scan?”	Explained radiological signs in lay language
High	“Patient has hemoptysis and history of TB—what does it mean?”	Provided cautious, informative reply with referral recommendation

### Rejection handling

One of TBAid’s most critical safety features is its ability to politely reject irrelevant or out-of-domain queries—a requirement in any [28] clinical chatbot system to reduce hallucinations or misinformative responses.

Rejection logic is enforced at two levels:

• Frontend rule filtering via keyword matching (e.g., rejecting queries like “How to lose weight?”)
• Backend Hugging Face prompt constraints using the Qwen/Qwen2.5-72B-Instruct model to limit LLM responses strictly to TB-related intents only

Table 6 illustrates how the chatbot handles out-of-domain queries using this two-tiered logic

**Table 6:** Rejected queries and system response examples.

User Query	Chatbot Response
“What are symptoms of a heart attack?”	“I'm sorry for any confusion, but as Aura, my expertise is strictly limited to Tuberculosis. I cannot provide information about symptoms of a heart attack. If you have concerns about heart attack symptoms, please consult a qualified healthcare provider immediately.On the other hand, let me share some information about the symptoms of Tuberculosis (TB): • Persistent coughing that lasts for more than three weeks • Coughing up blood or phlegm • Chest pain, or pain in the sides that won't go away • Unexplained weight loss • Fever • Night sweats • Fatigue • Loss of appetite If you or someone you know exhibits these symptoms, it is important to consult a healthcare provider for proper diagnosis and treatment. Again, I must stress that I am an AI specialized in Tuberculosis and not equipped to provide information about other medical conditions, such as heart attacks. Please consult a healthcare professional for accurate information.”
“How to treat diabetes?”	“I'm sorry for any confusion, but as Aura, my expertise is strictly limited to Tuberculosis. I cannot provide information on the treatment of other medical conditions like diabetes. It is important to consult a qualified healthcare provider for advice on treating diabetes. They can provide a personalized treatment plan based on your specific circumstances and condition.Some general information about diabetes, though, is that it is a chronic condition that affects how your body uses glucose (a type of sugar) for energy. There are three main types of diabetes – Type 1, Type 2, and Gestational Diabetes. The primary treatment generally involves lifestyle changes, oral medication, or insulin therapy.Lifestyle changes might include: 1. Healthy eating: Following a diet low in sugar, fat, and calories but high in fiber and nutrients. 2. Physical Activity: Regular exercise can help manage blood sugar levels more effectively. 3. Regular check-ups: Regular doctors' appointments to monitor blood sugar levels, blood pressure, and cholesterol levels. Always consult a healthcare provider for a proper diagnosis and treatment plan for diabetes. Do not attempt to self-diagnose or treat diabetes based on this general information.”
“Can I take vitamin D daily?”	“I'm sorry, but as Aura, my expertise is strictly limited to Tuberculosis. I cannot provide general health advice about taking vitamin D. However, it is worth noting that some scientific studies suggest that vitamin D supplementation could potentially play a role in the prevention of Tuberculosis by modulating immune responses and reducing inflammation. However, it is essential to consult a healthcare provider before starting any new supplement regimen, especially for long-term use, as they can provide personalized advice based on your health status and other medications you might be taking.You can find more information about vitamin D and its potential health benefits here: • Official government websites of your region (US) • NHS website (UK) • Canada's Food Guide (Canada)”

**Fig. 5:**
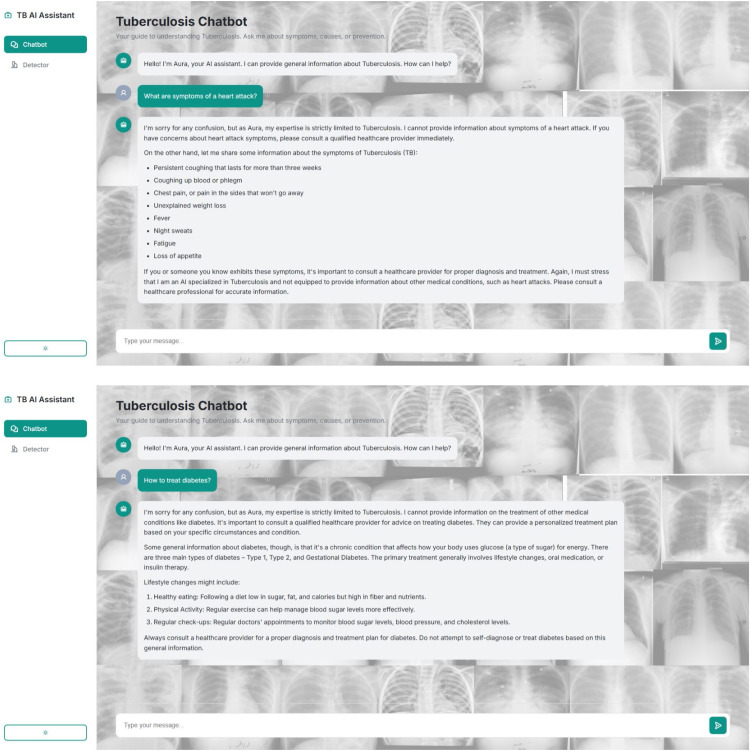
Controlled message rejection for off-domain query input.

As per Fig. 5, the chatbot's safety guardrails are demonstrated through its response to out-of-scope medical questions. The system correctly identifies queries about unrelated conditions, such as symptoms of a heart attack and treatment for diabetes, and politely declines to provide specific information. By explicitly stating its expertise is confined to tuberculosis and advising the user to consult a healthcare professional, the chatbot maintains its specialized focus and ensures user safety by avoiding the spread of unverified medical advice.

Such behavior of domain rejection gains trust and makes sure that the chatbot does not spread false or out-of-scope medical advice. It is also in line with views proposed in the literature before regarding safe deployment of AI chatbots in healthcare [29].

The functional analysis of TBAid with 120 benchmark queries indicates that TBAid is an effective resource as domain-limited patient-facing chatbot assistant in the area of tuberculosis education and support. The system was of high level in query classification and generating the response and the overall accuracy of the combined accuracy was 97.9 percent and it handled complex language inputs of both technical and non-technical users very effectively.

One of the main advantages of TBAid is that there is a high degree of domain restriction, the chatbot deals with questions about TB but refuses to answer out-of-topic questions politely and safely in terms of medical care. This choice of design tackles one of the primary risks of general-purpose medical chatbots, namely scope drift, i.e. systems trying to answer questions outside of their training, leading to misleading and dangerous recommendations.

Recent studies have demonstrated that artificial intelligence–based tuberculosis screening systems, particularly those leveraging chest X-ray (CXR) imaging, can achieve strong diagnostic performance while remaining computationally efficient and suitable for deployment in low-resource environments. Lightweight deep learning models such as LightTBNet have shown that carefully optimized convolutional architectures can deliver high accuracy, F1-score, and area under the ROC curve while maintaining minimal computational and memory requirements, enabling real-time inference on handheld or edge devices commonly used in high-TB-burden regions [30]. Broader reviews of AI-assisted TB diagnosis further highlight the potential of CXR-based systems to bridge diagnostic gaps in developing countries, where access to advanced imaging and specialist interpretation is limited [31]. Large-scale clinical evaluations comparing commercial AI algorithms with expert radiologists have also demonstrated that multiple CXR-based AI systems can meet or exceed World Health Organization triage benchmarks, significantly reducing the need for confirmatory molecular testing while maintaining high sensitivity [32]. In pediatric populations, where tuberculosis diagnosis is particularly challenging due to atypical presentations, multi-view CXR-based deep learning frameworks have shown robust performance across age groups and external datasets, reinforcing the clinical relevance of accessible radiographic modalities in resource-constrained settings [33]. These findings collectively underscore the importance of modality-appropriate, efficient, and explainable AI systems, complementing the role of TBAid as a domain-restricted conversational layer designed to translate both CXR- and CT-based diagnostic outputs into safe, non-expert–accessible explanations.

## Summary of evaluation findings

The evaluation demonstrates that TBAid consistently delivers accurate and clinically safe responses across varied query types while maintaining strict domain restriction. The consolidated accuracy results (low: 100 %, moderate: 97.5 %, high: 94.2 %, rejection: 100 %) reflect reliable handling of both clinical and non-expert user queries. These outcomes confirm the system’s effectiveness as a domain-restricted educational support tool for TB awareness.

**Fig. 6:**
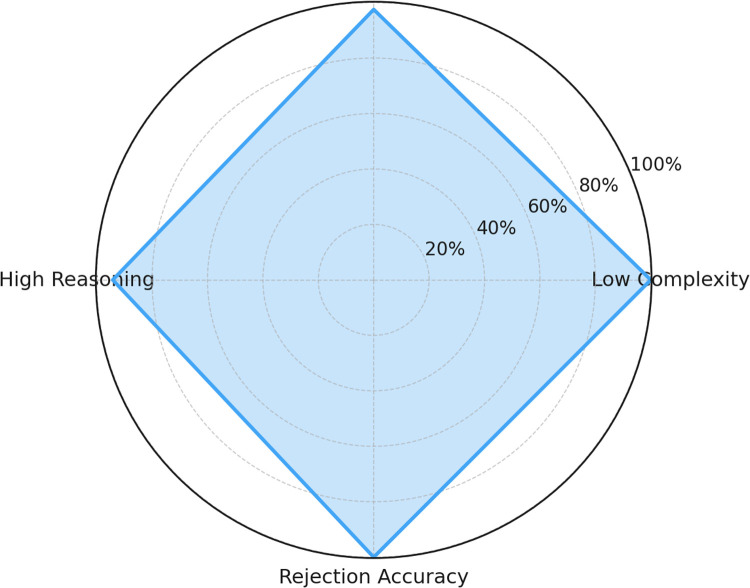
Radar chart – TBAid evaluation across query types.

The radar chart of Fig. 6 emphasizes the mixed accuracy of TBAid in a number of major areas, resulting in effective performance in addressing the subject of the attention:

• Low complexity queries: 100 %
• Moderate queries: 97.5 %
• High complexity reasoning: 94.2 %
• Rejected queries: 100 % rejection accuracy

The above implies that the chatbot is consistent in performance along a continuum of user intentions, including recreationalists and trainees in the medical field.

Another important metric for real-world usability is response latency. Across all evaluated queries, the median response time was 4.4 s, including frontend input, Flask backend routing, and Hugging Face Inference API response time for the Qwen/Qwen2.5–72B-Instruct model. This ensures that TBAid can be used in real-time educational or triage scenarios without disrupting clinical flow or delaying information access.

**Fig. 7:**
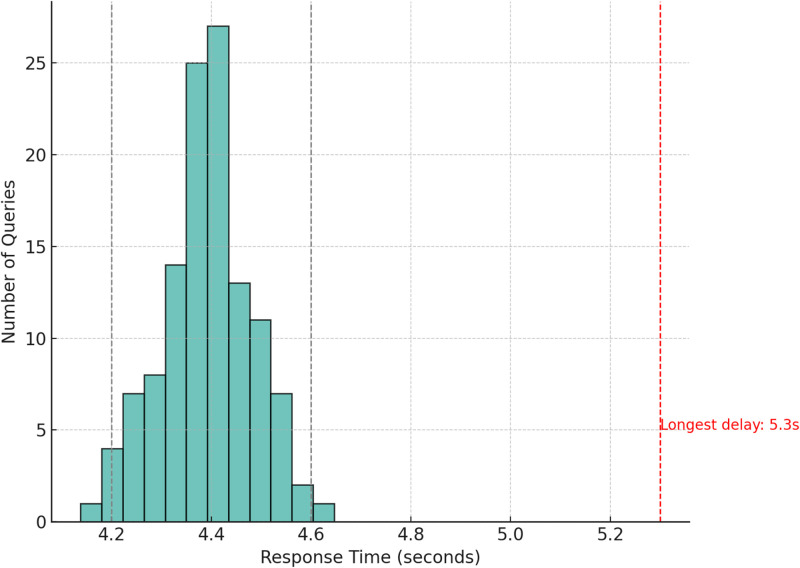
TBAid response time distribution (in seconds).

Fig. 7 displays a histogram of the system's response time distribution, measured in seconds:

• 85 % of responses fell between 4.2 – 4.6 s
• Longest delay observed: 5.3 s (due to network fluctuation)

While the current chatbot does not yet process CT scans directly, the parallel image classifier offers a viable pathway toward future integration. The planned feature to summarize binary TB predictions (“Detected” / “Not Detected”) in human-readable format will bridge the gap between AI outputs and patient understanding. This would be especially impactful in rural or primary care centers where radiology turnaround times may delay treatment.

Additionally, future enhancements—such as support for Hindi, Punjabi, and other regional languages—will expand accessibility and inclusivity, enabling frontline health workers and patients in under-resourced settings to benefit from intelligent, conversational support tools.

## Ethical considerations and risk analysis

Given that TBAid operates in the medical domain, a thorough consideration of ethical and safety implications is paramount.

• **Data Privacy:** The current implementation of TBAid is stateless; it does not store user queries, conversation histories, or any personally identifiable information. For any future deployment in a clinical setting, the system would be architected to comply with data protection regulations such as HIPAA, employing end-to-end encryption for all communications.
• **Risk of Misinformation and Misdiagnosis:** The primary risk of any medical chatbot is providing incorrect or misleading information. We mitigate this in three ways:
  ○ **Strict Domain Restriction:** As detailed in the methods, our dual-layer validation system (keyword filtering and constrained LLM prompting) ensures the chatbot does not answer out-of-scope questions.
  ○ **Disclaimer:** The chatbot's interface includes a persistent, clear disclaimer stating that it is an AI assistant, not a medical professional, and its guidance should never replace a consultation with a qualified doctor.
  ○ **Non-Diagnostic Role:** The system is explicitly designed for awareness and support, not for diagnosis. Even with future CT-model integration, its role is to translate technical results into understandable language, not to provide a diagnosis.
• **Countermeasures for Unsafe Outputs:** AI hallucinations are a known risk in LLMs. Our strict domain restriction is the primary countermeasure, as it severely narrows the scope of possible responses and prevents the model from generating information on unrelated topics, which is a common cause of unsafe outputs.

## Limitations

Despite the promising performance of TBAid, the system has certain limitations. Currently, it does not support real-time image interpretation, as integration with the CT-based classification model is still under development. The chatbot operates strictly within the tuberculosis domain and cannot handle comorbid conditions or general medical queries, which may restrict its utility in real-world scenarios where multiple health issues coexist. Moreover, the responses are rule-based and may lack adaptability in handling nuanced or context-rich queries. Lastly, the current version supports only English, limiting accessibility for non-English-speaking users in rural and underserved regions. Furthermore, the validation was performed using a benchmark set of 120 structured queries. While this allowed for a controlled evaluation of the system's accuracy and domain-restriction capabilities, it does not fully represent the diversity and unpredictability of real-world conversational inputs. The system's generalizability to colloquial language, slang, or fragmented user queries has not been statistically validated in a large-scale study. Future work will unify both CXR- and CT-based pipelines under a single chatbot interface with modality-adaptive explanations, while also conducting in-field usability testing with real patients and healthcare workers and expanding evaluation beyond structured queries to include real-world and colloquial inputs, ensuring robustness and clinical effectiveness.

**Multilingual Expansion Strategy**: Future development of TBAid includes multilingual deployment to improve accessibility for diverse patient groups. The planned approach combines a lightweight **translation-middleware layer** (for regional languages such as Hindi, Punjabi, and Bengali) with **domain-specific prompt tuning** to preserve medical meaning and terminology accuracy. The middleware converts user input into English for LLM processing and then returns context-preserved translated output. This phased strategy avoids loss of clinical semantics and enables scalable multilingual support without modifying the core architecture.

## Ethics statements

This research did not involve human participants, animal experiments, or data collected from social media platforms. All data utilized in this study were collected by researchers adhering to the respective ethical guidelines and without violating privacy rights. No additional ethical approval was required for the use of these datasets in our study.

## Consent of the patients

We would like to clarify that no patient-specific or personally identifiable medical images were used in this study. All images referenced or utilized (e.g., chest X-rays) were sourced from open-access, publicly available datasets that are ethically cleared for research and educational purposes. These images are not linked to any identifiable individuals, and their use complies with the respective dataset guidelines.

## Acknowledgments

*We thank the Management, Director, HODs, and staff of SIU for their support and guidance*

*This research did not receive any specific grant from funding agencies in the public, commercial, or not-for-profit sectors.*

## CRediT authorship contribution statement

**Lakshmi Ravi Teja Meka:** Visualization, Investigation. **Dalwinder Singh:** Data curation, Writing – original draft. **Arun Singh:** Software, Validation. **Saiprasad Potharaju:** Conceptualization, Methodology, Software. **M.V.V. Prasad Kantipudi:** Supervision. **Swathi Gowroju:** Writing – review & editing.

## Declaration of interests

The authors declare that they have no known competing financial interests or personal relationships that could have appeared to influence the work reported in this paper.

## Data Availability

Data will be made available on request.
